# Integrative illustration of a JCVI-syn3A minimal cell

**DOI:** 10.1515/jib-2022-0013

**Published:** 2022-06-27

**Authors:** David S. Goodsell

**Affiliations:** Department of Integrative Structural and Computational Biology, The Scripps Research Institute, La Jolla, CA 92037, USA; Research Collaboratory for Structural Bioinformatics Protein Data Bank, Rutgers, The State University of New Jersey, Piscataway, NJ 08854, USA

**Keywords:** cellular structure, molecular modelling, proteome, science education and outreach, synthetic cell

## Abstract

Data from genomics, proteomics, structural biology and cryo-electron microscopy are integrated into a structural illustration of a cross section through an entire JCVI-syn3.0 minimal cell. The illustration is designed with several goals: to inspire excitement in science, to depict the underlying scientific results accurately, and to be feasible in traditional media. Design choices to achieve these goals include reduction of visual complexity with simplified representations, use of orthographic projection to retain scale relationships, and an approach to color that highlights functional compartments of the cell. Given that this simple cell provides an attractive laboratory for exploring the central processes needed for life, several functional narratives are included in the illustration, including division of the cell and the first depiction of an entire cellular proteome. The illustration lays the foundation for 3D molecular modeling of this cell.

## 1 Introduction

In the early 1990s, I posed a hypothesis: “Is it possible to create an illustration of a living cell that shows all macromolecules, drawn to scale and integrating available structural and biophysical information?” I chose *Escherichia coli* as the subject, and at the time, the answer to this question was “Almost”. At the time, the Science Citation Index was the primary resource for searching the scientific literature, requiring a laborious process of tracking down one piece of information at a time. Proteomic information was primarily available as lists of proteins identified one-by-one from 2D gel electrophoresis. The Protein Data Bank (PDB) archive contained about 300 structures and electron microscopy provided only the general contours of large assemblies. Finding information on the abundance of molecular components was a particularly challenging aspect of my literature search. Since the genome had not yet been determined, I also relied on studies of molecular weight distributions and presumed oligomerization states to fill in a representative collection of soluble enzymes. I created three 100 nm square cross-section images (cell wall, cytoplasm and nucleoid) [1], reflecting the state of knowledge at the time, tempered, of course, by my ability to find this information. In the years since then, I have continually updated these illustrations, both for integrating new information to reflect the current state of knowledge and for refining the design to improve clarity.

Today, the answer to this question is a slightly-nuanced “Yes”. My most recent *E. coli* illustration (doi: 10.2210/rcsb_pdb/goodsell-gallery-028) is now based on a genome, a proteome with abundances, and a flood of new structural information from the revolution in cryoelectron microscopy [2]. This information is complemented by powerful tools to find it, such as the GenBank [3], UniProt [4], PDB [5], and PubMed (pubmed.gov), greatly streamlining the process of creating of integrative works.

**Figure 1:**
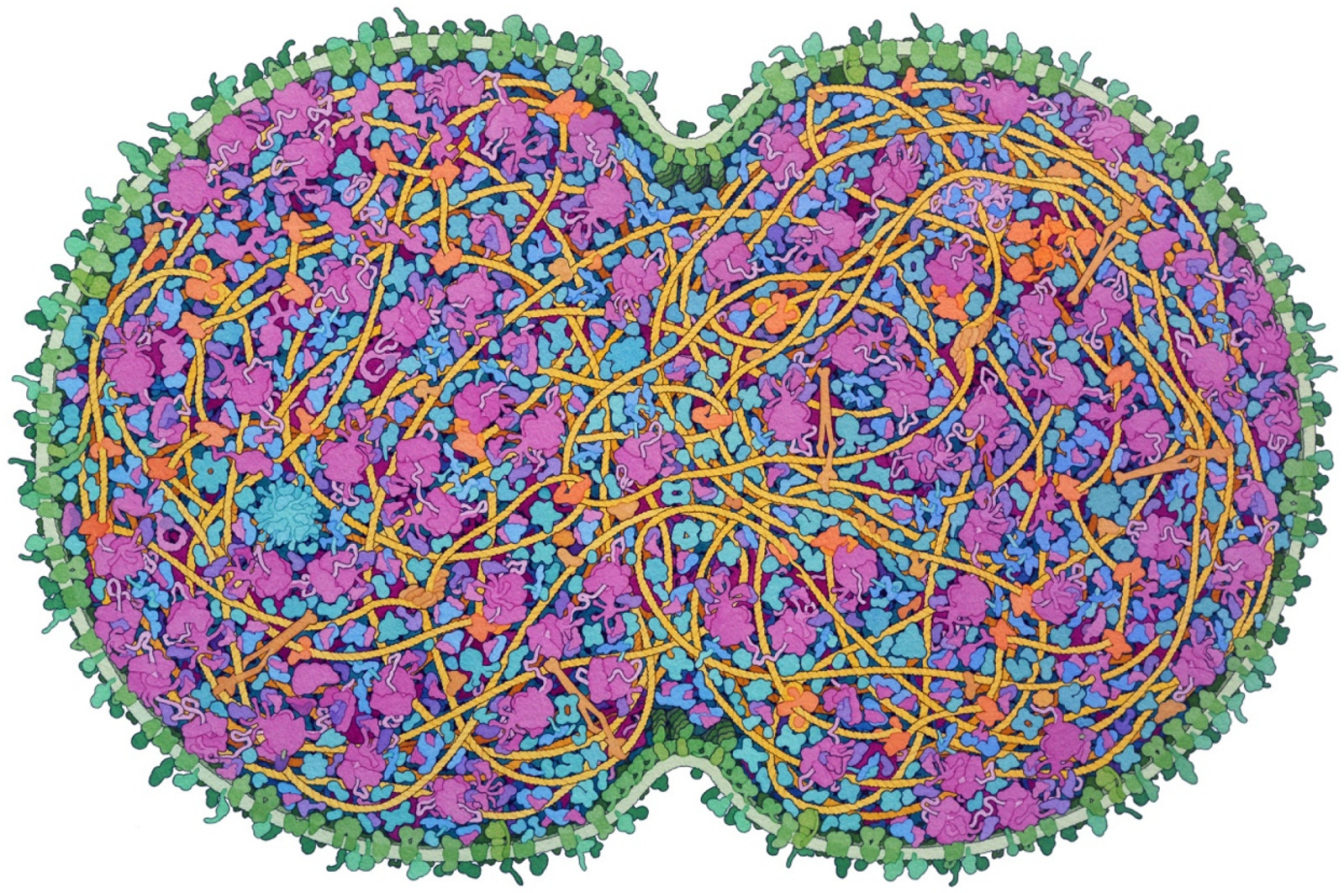
Artistic conception of a cross-section through a dividing JCVI-syn3A cell. Small molecules, ions, and water are omitted for clarity.

 The JCVI-syn3A cell provides a unique opportunity: to create an illustration that depicts an entire proteome of a living cell. Syn3A is the culmination of work at the J. Craig Venter Institute to create a synthetic cell that includes a minimal number of genes to support life. The cell was developed in several steps, starting from a *Mycoplasma mycoides* cell with a synthetic genome (syn1.0), and then paring away non-essential genes in successive rounds of reduction [6]. In the most recent step, from syn3.0 to syn3A, a handful of genes were added back to improve the stability of cell division [7]. Because of its minimal nature, syn3A is an attractive model organism for experimental study, and as such, a large amount of experimental information is available for its structure, ultrastructure, and metabolism. In this report, I describe the integration of this data to create an illustration of a cross-section through a syn3A cell, showing all macromolecules to scale (Figure 1).

## 2 Design choices

I have several overarching goals for these paintings, which together guide my design choices for color and representation [8]. Foremost, I want to create images that are appealing and interpretable by non-expert viewers. Second, I want to stay true to the science to give an accurate impression of the sizes, shapes and distributions of molecules in the cellular environment. Finally, I need a stylistic approach that is feasible within the limited time that I have for artistic creation. Of course, I do this work as part of a rich and diverse SciArt and visualization community, and I am a voracious consumer of scientific imagery. I continually take inspiration from the many compelling approaches that I find. Care must be taken, however, when comparing and applying these approaches, since they often have very different goals. For example, current trends in scientific data visualization are advocating subdued color palettes, where vibrant color is used sparingly to highlight only select features. This approach to color is quite different from my brightly colored approach, given that my primary goal is to generate excitement.

As in most of my previous work, I chose to include only macromolecules, since these cellular illustrations become intractable when small molecules are included. I typically create illustrations at a consistent magnification of 2,000,000X, which is often reduced by about two-fold when printed or presented online. This magnification is sufficient to show a scene several hundred nanometers wide on a typical printed page while including enough detail that the molecular shapes are recognizable. The size of this cell, however, necessitated rendering the original watercolor illustration at 1,000,000X, which allowed depiction of the entire cell on standard Arches watercolor paper.

Everything is drawn in orthographic projection, so that the sizes and shapes of all molecules are directly comparable across the image, with no perspective distortion. This is a marked departure from most molecular viewers, which typically display perspective views that are more compatible with the wide range of global and close-up views that are possible with interactive zooming. The illustration, on the other hand, depicts a static, panoramic view of a thin slab of the cell. Since the view is static, sharp depth cueing to black and slight overlapping of neighboring molecules is used to accent depth relationships.

I allow abundant artistic license to improve the interpretability of the image [9]. This includes careful placement of DNA and other extended molecules so that none are clipped by the cross-section, and placement of membranes to give appealing contours when clipped. Molecules are oriented with canonical views to allow easy recognition and showing interesting symmetry and interaction relationships. Molecular shapes are simplified, typically only showing overall form and subunit relationships, to help focus attention on the overall scene rather than the atomic details of each component. In addition, the level of magnification in the original painting required a slight exaggeration of the width of RNA and disordered protein chains, so that they would not be lost in the ink outlining.

The color palette is chosen to highlight the functional categories of each molecule [10]. This coloring scheme is consistent with my previous work, allowing direct comparison with other integrative illustrations. Given the primacy of the genome, DNA is in bright yellow, DNA-associated proteins are in tan, and DNA-associated enzymes (polymerases and topoisomerases) are in orange. The membrane is in green, with membrane-spanning proteins in a more saturated green and lipoproteins in bluer green. RNA, including ribosomes, tRNA and mRNA, are in shades of magenta, and protein synthesis factors are in purple. Enzymes are in shades of blue, with a cooler cobalt blue for enzymes interacting with the protein synthesis machinery, and metabolic enzymes in shades of turquoise.

Finally, in these types of illustrations, I made the decision to be agnostic about the source of information when designing the image. I make no attempt to depict confidence levels or sources for each component, for example, by tuning the representation based on the experimental resolution of each structure. Rather, once I have the best information I can find for each component, I render the entire scene in a consistent style at a consistent level of detail. This choice is designed to simulate a snapshot of the cell, rather than present how each component has been studied. We are currently exploring 3-D models of cells as a mechanism for display of this type of information, where representations, coloring schemes, and annotations are more readily changeable [11].

## 3 Narrative decisions

**Figure 2:**
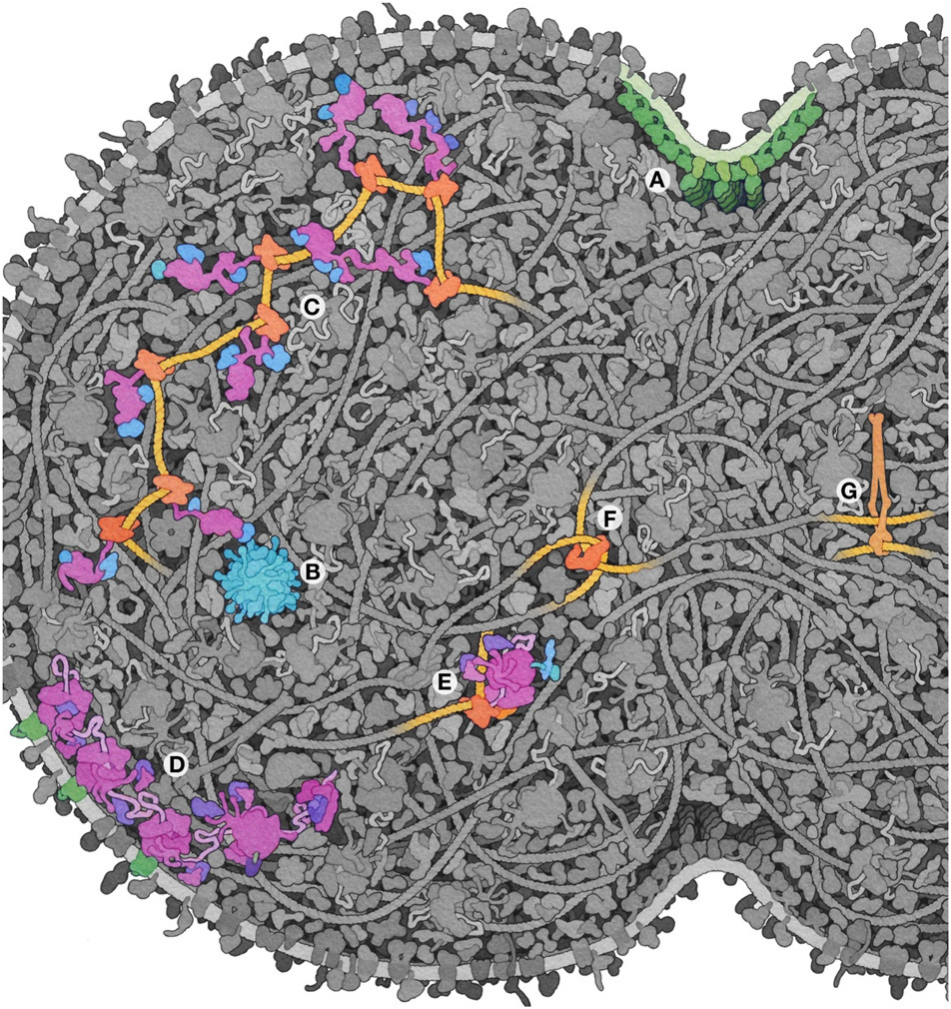
Narratives depicted in the illustration. (A) Proteins involved in cellular scission. (B) Pyruvate dehydrogenase complex, the largest assembly in the proteome. (C) Transcription of a ribosomal RNA operon. (D) Initiation and translation of membrane proteins by membrane-bound ribosomes. (E) An expressome with co-transcriptional translation. Two proteins managing the topology of chromosomes: (F) Topoisomerase and (G) Structural maintenance of chromosomes complex.

Once the major design choices are finalized, there is much freedom to tune the scene to focus attention on particular narratives [12]. The cell is depicted just after beginning division, to underscore the role of several cell division proteins (ftsZ, ftsA, and sepF), two of which are key elements included in the move from Syn3.0 to Syn3A (Figure 2A). Long filaments of ftsZ are thought to mediate cell division through interactions of membrane-binding proteins ftsA and sepF [13]. In order to highlight the shape of the cleavage furrow, the ftsZ filaments are depicted perpendicular to the plane of the page. Recent study of sepF indicates that it forms rings with the membrane-interacting surface on the inner face [14], so it is depicted as forming a C-shaped assembly cupping the membrane. Uncharacterized protein JCVISYN3A_0239 was predicted to have a single transmembrane segment and several spectrin repeats, and was included in a speculative interaction with ftsZ/ftsA similar to EzrA [15].

One overarching goal of this illustration is to depict an entire proteome. This is possible due to the limited size of the Syn3A genome, and the abundance of data that is currently available. The genome of Syn3A annotates 452 proteins and 38 RNA, plus two additional proteins needed for cloning in yeast (www.ncbi.nlm.nih.gov/nuccore/CP016816.2). After structure assignment, these yielded 329 molecular assemblies (see doi:10.2210/rcsb_pdb/goodsell-gallery-042 for a full list and key to the illustration) ranging in size from small acyl-carrier proteins to the huge pyruvate dehydrogenase complex (Figure 2B). Functional RNA (ribosomal subunits, tRNA, tmRNA, RNase P, and signal recognition particle) are also included with their protein partners in the assembly list.

Several features of ribosomes are highlighted in the illustration, based on current conceptions of ribosome structure and function. In both daughter cells, ribosomal RNA is being transcribed by multiple RNA polymerases from rRNA operons (Figure 2C). Ribosomes are depicted with L7/L12 stalk proteins associated with EF-G and EF-Tu/tRNA complexes [16], and in some cases, are further associated into polysomes. Several ribosomes are also shown associating or associated with transport channels at the cell surface [17] (Figure 2D). Given the presence of antitermination proteins nusG and nusA in the genome, several expressomes are included [18] (Figure 2E).

The illustration is also built around several narratives about the nucleoid. The illustration assumes entropic segregation of the daughter DNA strands [19] and depicts two topoisomerases resolving entanglements (Figure 2F). Many additional nucleoid-associated proteins are also depicted, including the structural maintenance of chromosomes (SMC) complex (Figure 2G), regulatory proteins and DNA repair enzymes.

## 4 Structures and abundances

The illustration builds extensively on recent experimental and modeling work on Syn3A, including systems biology modeling of metabolic and genetic networks and structural modeling with LatticeMicrobes [7, 20], [21], [22]. That work reconciles the identity and abundance of gene products found in the proteome study [7], and provided a touchstone throughout researching and creating the illustration to define the molecular composition of the cell. Features from those studies that were included in the illustration include the ∼400 nm diameter of the spherical cell, lack of supercoiled plectonemes in the nucleoid, random distribution of ribosomes, and presence of expressomes. The gene location, gene name, locus tag, protein name, and protein id were extracted for gene products and non-coding RNA from the genome. Abundances were averaged for the three time points reported in the proteome, and for assemblies, abundances for all subunits were averaged. Arbitrary abundances of 20/cell were assigned for the proteins missing from the proteome. Cell surface lipoglycans were not included after a personal communication with mycoplasma expert James Daubenspeck (University of Alabama at Birmingham, USA).

Compilation of the structural proteome leveraged the recent whole-cell structural model of *Mycoplasma genitalium* [11]. Structural homologs in the *M. genitalium* proteome were found in several successive steps. Many were assigned by matching gene names in UniProt. If no match was found, a BLAST search was performed at NCBI using the default parameters. In cases where this was not successful, manual search of the protein name in UniProt and RCSB Protein Data Bank was performed.

Most of the structures from the *M. genitalium* structural proteome were used as-is, however alternative decisions were made in several cases. For proteins interacting with tRNA (such as amino acid--tRNA ligases and EF-Tu), the protein is depicted as a complex with tRNA. Ribosomes in multiple states are depicted similarly to previous work with *E. coli* (doi: 10.2210/rcsb_pdb/goodsell-gallery-028). Several complex DNA-associated proteins were based on published reports: DNA gyrase [23], DNA topoisomerase IV [24], and RNA polymerase and the expressome [18].

**Figure 3:**
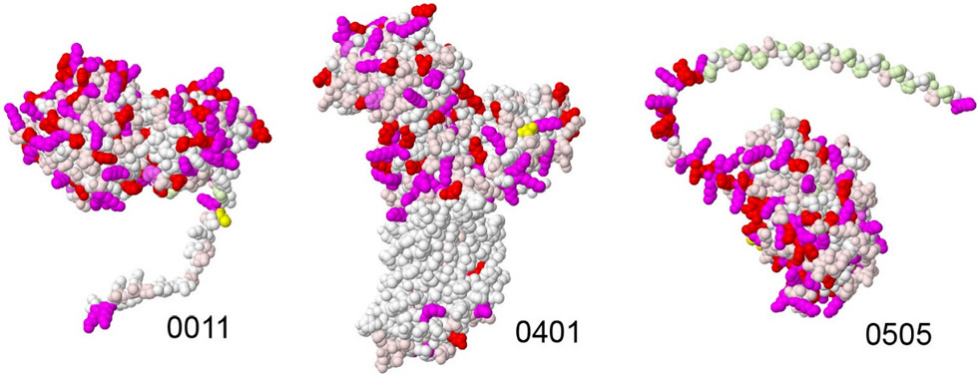
Coloring scheme used for curation of AlphaFold2 structures. This scheme highlights hydrophobic regions (white) versus charged amino acids (red and magenta). Amino acids with specific functions are given unique colors: cysteine in yellow and proline in light green. JCVISYN3A_0011 is a lipoprotein with a characteristic unfolded signal sequence. It will be clipped off after transport out of the cell and lipidated at the cysteine at the top of the signal sequence. 0401 shows a prominent hydrophobic belt, which is the presumed membrane-spanning portion. 0505 has a long proline-rich tail predicted to be intrinsically disordered.

**Figure 4:**
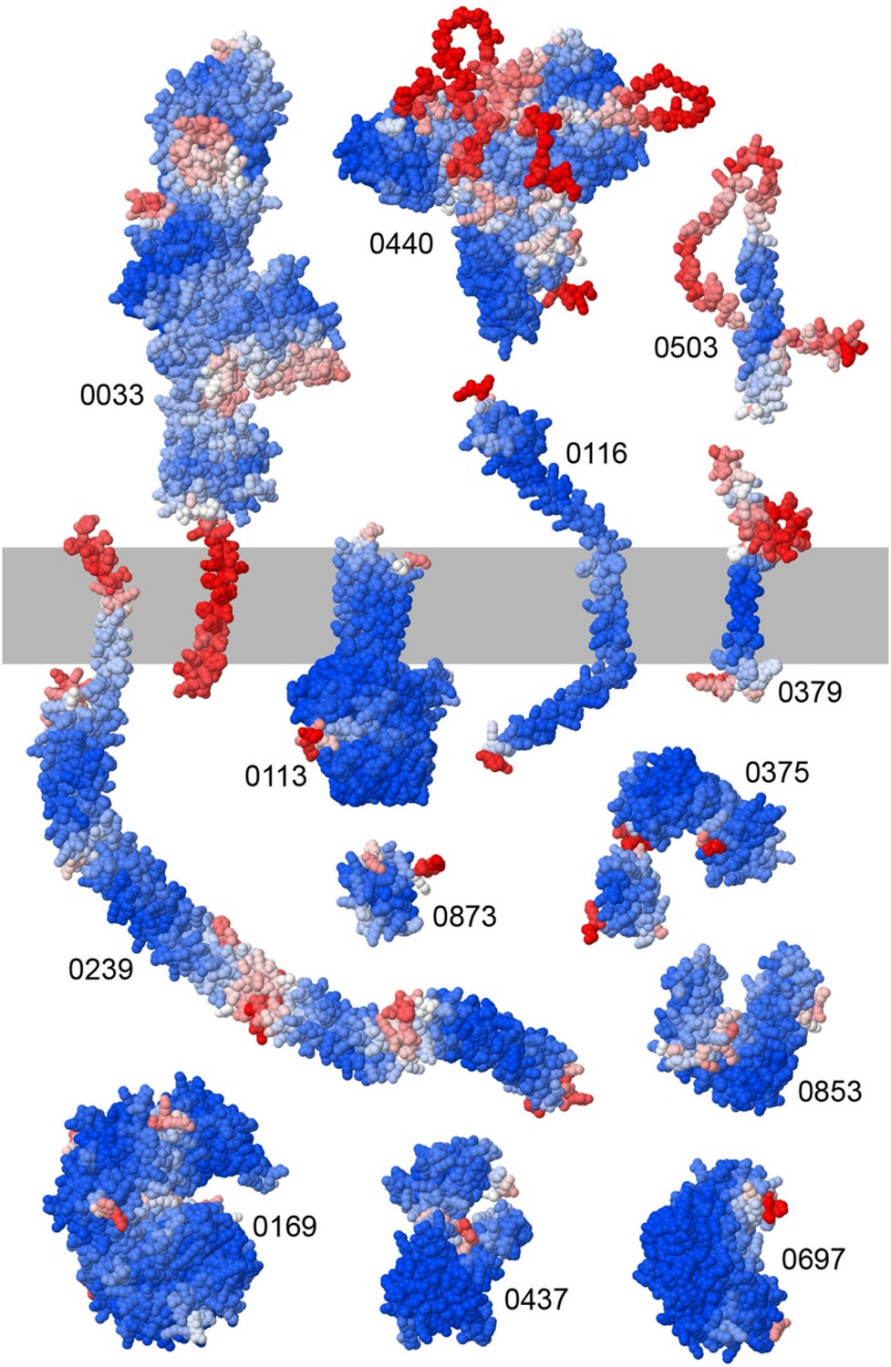
Selected AlphaFold2 structures. Each structure is colored with regions of high confidence in blue and low confidence in red. Many membrane proteins gave credible predicted structures: 0239 has a membrane-spanning segment connected to several spectrin-like repeats. 0033 is an “STREFT” (secreted thousand-residue frequently tandem) lipoprotein characteristic of mycoplasmas and related cells. 0113 was annotated as a glycolipid synthase and has an enzyme-like domain extending from the inner side of the membrane. Some smaller membrane proteins, such as 0116 and 0379, did not give folded structures and are presumed to be part of larger assemblies. Many soluble proteins adopted compact folds, such as the small protein 0873 and larger proteins 0169, 0437, and 0697. Some of these showed multiple domains that may be part of larger assemblies, such as 0375 and 0853. Finally some proteins failed to fold, such as 0440 and 0503.

Structural homologs were not found for 89 proteins, most of which are annotated as “uncharacterized proteins” in the genome. Structures were predicted with AlphaFold2 [25]. These were then manually examined in Jmol [26] using a representation that highlights surface hydrophobicity, and compared with predictions of membrane-spanning regions from UniProt (Figure 3). Useable predictions were obtained for most of the proteins (see examples in Figure 4). For membrane-bound proteins, the predicted structures matched the UniProt annotations, and membrane portions showed as a prominent hydrophobic alpha helix or a hydrophobic belt around globular proteins. Lipoproteins were modeled with signal sequences, and in all cases, the signal sequence was predicted to be disordered, and was removed for the final model and replaced by lipidation. Fourteen gene products are small chains that did not fold into a globular structure, and were omitted from the illustration. These are assumed to be subunits of speculative larger assemblies.

## 5 Building and rendering the illustration

The illustration was executed using previously reported techniques [8]. Briefly, style sheets for all molecules were created at a consistent magnification and used to develop a full sketch of the foreground of the scene. Abundances were approximated in the following manner: ribosomes were added first based on distribution in cryoEM images [21] and the nucleoid sketched around them; multiple copies of abundant translation factors, tRNA, chaperones, and metabolic enzymes were then added; the remaining space was filled with one or two copies of less abundant proteins to complete the proteome. This foreground sketch was transferred to Arches 300 lb Rough Natural White paper using carbon paper. Color was added using a watercolor palette mixed largely from Windsor and Newton cadmium yellow, yellow ochre, Windsor red, viridian hue, cobalt blue, and Old Holland magenta. Background molecules were then added extemporaneously, depth-cued to darker shades using Vandyke brown for warm colors and ivory black for cool colors. Outlines were rendered in India ink with a 00 (0.3 mm) Rapidograph technical pen.

## 6 Applications

My illustrations of bacterial cells are widely used to depict the crowded nature of cells, often being used as introductory figures in textbooks, professional presentations, and popular media. The syn3A illustration is already being used, for example, as the introductory artwork for an article about synthetic biology in *New Yorker* magazine. In this capacity, I hope that the illustration will help inspire wonder at the complexity of these so-called “simple” cells. My own sense of wonder continues to grow with each scene that I render, as I explore new mechanisms as they are revealed by experimentalists. For example, in this illustration, I was able to work through an entire cellular proteome and ponder on the structure and function of every protein, and marvel at how few proteins are actually necessary to support the life of this cell.

**Figure 5:**
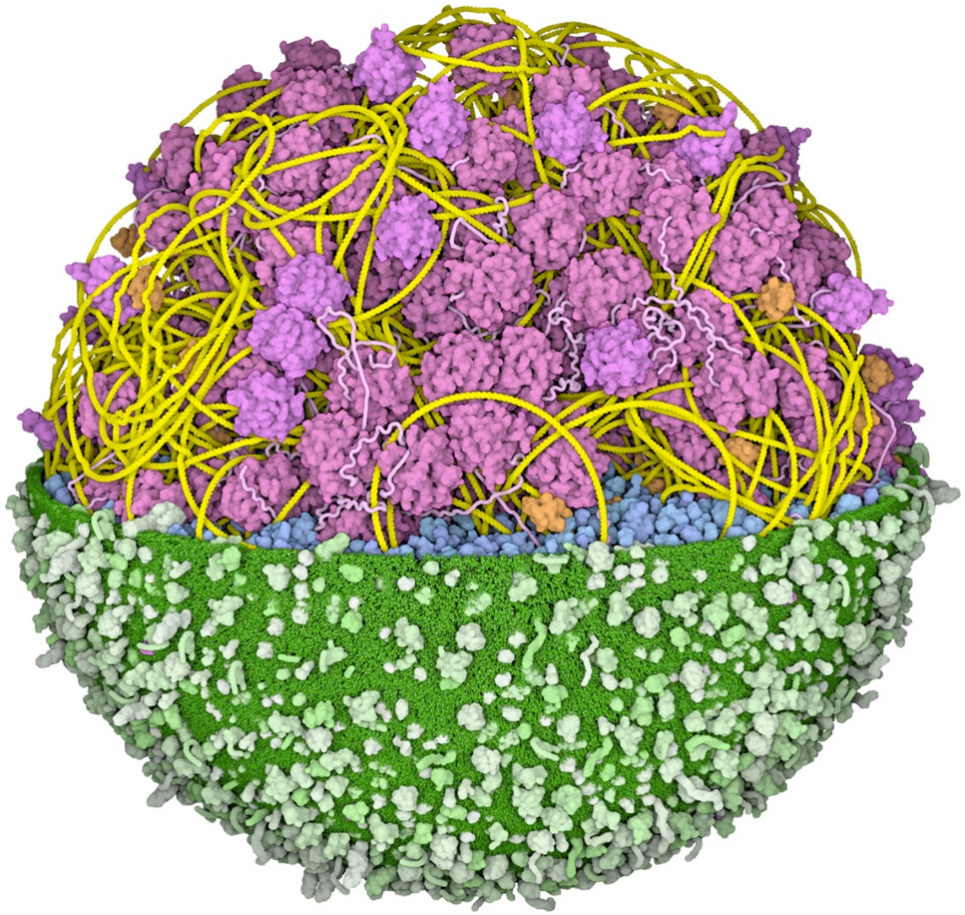
3D whole cell model of syn3A minimal cell. The upper half is clipped away to show the DNA, polymerases, mRNA, and ribosomes. Image created by Ludovic Autin.

As computational and bioinformatics hardware and software continue to improve, I am also increasingly presenting these illustrations as a challenge to the field of 3D mesoscale modeling. In our own work on modeling bacterial cells, I often create these types of illustrations as a way to identify structural features that require development of new modeling techniques. This was apparent in the syn3A illustration: many aspects of syn3A structure are relatively easy to depict in an illustration, but still pose great challenges for modeling. For example, we have recently created a draft model of a syn3A cell using the CellPACK suite of modeling tools (Figure 5) [11, 27, 28]. These tools are effective for creating the basic structure of the cell, including: a membrane with embedded proteins; a nucleoid with associated polymerases, mRNA, ribosomes, and nucleic-acid-binding proteins; and a collection of soluble cytoplasmic proteins. However, current challenges include: modeling of replication intermediates and segregation of the chromosome; modeling of the machinery of cell division; modeling intrinsically-disordered proteins such as the ribosomal L7/L12 stalk proteins and pyruvate dehydrogenase complex; and an effective way of identifying and modeling transient complexes that would be expected to be present at any given time point in the cell cycle. Illustration provides a relatively nimble method to explore ideas for assembling and depicting these high-order structure/function features, to inform the more time-intensive structural modeling development effort.

## 7 Prospects

As you might expect, the design approach described here relies on my own experience in drawing and watercolor painting, and thus may not be accessible to most researchers. We are building new turnkey tools to address this challenge, to allow a wide community of users to create these types of cellular illustrations without need for artistic training in traditional media. Our program CellPAINT [29] works like a digital painting program with molecules as brushes that may be painted into a larger cellular scene. CellPAINT is designed with default coloring and representation that build on the design approaches described here. In my experience, researchers often do not have the time or resources to focus on these types of conscious design choices, and often rely on these types of defaults in the software that they use to visualize their results. However, I feel that one of the contributions of my own work is to provide worked examples of how deliberate design choices can help create and tune visualization approaches for new scientific domains. In CellPAINT, we have built in many options for customization of color and representation to foster additional creativity from users. I am also increasingly presenting design reports such as this article in scientific settings, with the goal of encouraging other researchers to spend time thinking about their own design decisions and hopefully to build new and personal visual languages in their own domains.

## 8 Availability

The illustration and a detailed key are freely available under a Creative Commons CC-BY-4.0 license at PDB-101, the outreach and education portal of the RCSB Protein Data Bank: doi:10.2210/rcsb_pdb/goodsell-gallery-042.
